# Thirty-day hospital readmission prediction model based on common data model with weather and air quality data

**DOI:** 10.1038/s41598-021-02395-9

**Published:** 2021-12-02

**Authors:** Borim Ryu, Sooyoung Yoo, Seok Kim, Jinwook Choi

**Affiliations:** 1 Office of eHealth Research and Business, Seoul National University Bundang Hospital, 172, Dolma-ro, Bundang-gu, Seongnam-si, 13605 Gyeonggi-do Republic of Korea; 2 Department of Biomedical Engineering, College of Medicine, Seoul National University, 28 Yongon-Dong Chongro-Gu, Seoul, 110-799 Korea; 3 Institute of Medical and Biological Engineering, Medical Research Center, Seoul National University, Seoul, South Korea

**Keywords:** Biomedical engineering, Risk factors, Machine learning

## Abstract

Although several studies have attempted to develop a model for predicting 30-day re-hospitalization, few attempts have been made for sufficient verification and multi-center expansion for clinical use. In this study, we developed a model that predicts unplanned hospital readmission within 30 days of discharge; the model is based on a common data model and considers weather and air quality factors, and can be easily extended to multiple hospitals. We developed and compared four tree-based machine learning methods: decision tree, random forest, AdaBoost, and gradient boosting machine (GBM). Above all, GBM showed the highest AUC performance of 75.1 in the clinical model, while the clinical and W-score model showed the best performance of 73.9 for musculoskeletal diseases. Further, PM10, rainfall, and maximum temperature were the weather and air quality variables that most impacted the model. In addition, external validation has confirmed that the model based on weather and air quality factors has transportability to adapt to other hospital systems.

## Introduction

Unplanned readmission after discharge is an indicator of quality of care, often suggests problems with patient care during previous hospitalizations, and is also a key indicator of the adequacy of a patient's treatment plan. Broadly defined, a hospital readmission involves the readmission of a patient who had been discharged from a hospital, to the same or another hospital within a specified time frame. The original hospital stay is often called the "index admission" and the subsequent hospital stay is called the "readmission." Different time frames have been used for research purposes, the most common being 30-day, 90-day, and 1-year readmissions. In the United States, it has been reported that 20% of Medicare patients are readmitted within 30 days of discharge, and that unplanned readmissions cost an estimated $17.4 billion. In the United States, as part of the Affordable Care Act at the national level, institutional mechanisms have been established, such as providing incentives for each hospital based on the readmissions within 30 days of discharge. Since then, various studies have been conducted on unplanned hospital readmissions within 30 days, and many countries are using it as an indicator of quality of care. Considering this, our study also aims to develop a system for predicting readmission within 30 days, which can be applied to actual clinical practice.

The relationship among climate, human health, and diseases has been established through multiple studies in the past^1^, and various risk factors for hospital admission have been studied based on demographic, environmental, and clinical factors^2–9^. Temperature variation due to global warming has been linked to hospital admission rates^10–13^. Humidity has been noted as another important health factor, while pollutants, including carbon monoxide and fine air particulates, have been found to be associated with increased admissions for multiple conditions^13–15^. Further investigation of the association between the ambient climate condition and hospital admission will help healthcare stakeholders understand the severity of the effect of weather change and implement healthcare-resource and patient-care plans.

The common data model (CDM) is a healthcare data model based on standard terminology. An example is the CDM developed by the Observational Medical Outcomes Partnership (OMOP) and maintained by the Observational Health Data Sciences and Informatics (OHDSI)^16–18^. A system developed by converting data into a CDM is easily applicable through the distribution of the source code of the program without the need for installing the software on a specific institution’s system^19^. CDM is a data model based on common standard terms. Therefore, it guarantees standardized content from the data model and exhibits high extensibility.

In the present study, we developed and validated four prediction models for hospital readmission within 30 days of discharge using the OMOP CDM as well as weather and air quality factors. In addition, the model performance was externally validated to examine its extensibility. To the best of our knowledge, the present study is the first to create a patient-level prediction model for hospital readmission within 30 days using OMOP CDM and ambient weather data. A predictive model that combines weather and environmental data with a patient’s residence information is expected to enhance clinical decision making at the individual patient level. More specifically, the W-score of an individual patient was obtained by adding up the forecast values for each weather element for 7 days from the date of discharge, which enabled the use of the weather forecast data of the Korea Meteorological Administration to predict the re-hospitalization of this patient at the time of discharge. The model was designed with a view to using the weather forecast data for the next 7 days for the patient's address for clinical decision-making.

## Results

Of the 61,922 index hospitalizations from the Seoul National University Hospital (SNUH) data included in our cohort research, 5794 resulted in a 30-day readmission through emergency-room visits (Table 1). The mean age of the readmitted individuals was 75.2 years, and more than half of the readmitted patients were males. The average length of stay was 2.5 days for the readmitted group and 0.2 days for the non-readmitted group.

**Table 1:** Basic characteristics of study data for each visit type.

Characteristics		Derived cohorts		P value
		Readmitted (N = 5794)	Non-readmitted (N = 56,128)	
Age, year, mean (SD)		75.2 (6.8)	74.7 (6.7)	0.000
Gender	Male, n (%)	54.8	49.7	
	Female, n (%)	45.2	50.3	
Age during hospital visit	60s	23.8	26.0	
	70s	49.7	50.2	
	80s	23.7	21.4	
	90s	2.7	2.4	
Season during admission	Spring	25.4	24.1	0.049
	Summer	25.5	26.8	
	Fall	24.4	24.8	
	Winter	24.6	24.3	
Average length of stay, mean (SD)		2.5 (4.3)	0.2 (0.4)	
Charlson comorbidity index, mean		1.11	0.52	

Table 2 presents the number of patient visits and readmission incidence rate in different disease groups. The internal and external validation results of the proposed readmission prediction models are presented in Table 3, where we can observe the differences in model performance among different diseases. The external validation results indicate that the proposed models show significantly improved performance for the musculoskeletal disease group. The main purpose of external validation is to verify how generalized and interpretable the developed model can be for performance evaluation of the developed model. In this study, it is expected that the model performance improved in the external validation experiment due to the difference between the size of the data used for the external validation and the size of the data for which the model was developed (and internal validation was performed). Since the results were better in the verification process of the model for larger data, we are confident that the model created in this study is robust enough for generalization. Furthermore, supplementary Table S3-S6 shows the top 20 predictors of each model in this study. According to Table 4, PM10, rainfall, and maximum temperature were the weather and air quality variables that most impacted the model among the disease groups.

**Table 2:** Number of visits in each disease group and outcome incidence rate in our research cohorts.

Disease groups	Train/test population (internal)		Valid population (external)	
	Target size (N)	% incidence	Target size (N)	% incidence
Diseases of the circulatory system (I00–I99)	9357	14.0	87,063	10.3
Mental and behavioral disorders (F00–F99)	3174	16.3	7228	17.5
Diseases of the musculoskeletal system and connective tissue (M00–M99)	13,564	11.8	41,015	11.7
Diseases of the respiratory system (J00–J99)	10,310	15.7	87,604	15.1

**Table 3:** Comparison of disease-specific performance in each model based on the area under the receiver operating characteristic curve.

Disease groups	Prediction models	Internal validation		External validation	
		Clinical covariates only	Clinical covariates and W-scores	Clinical covariates only	Clinical covariates and W-scores
Diseases of the circulatory system	DT	0.653	0.674	0.664	0.679
	RF	0.693	0.686	0.688	0.681
	ADA	0.698	0.708	0.672	0.67
	GBM	0.726^a^	0.717^a^	0.704^a^	0.696^a^
Mental and behavioral disorders	DT	0.612	0.691	0.706	0.737
	RF	0.703	0.692^a^	0.743	0.686^a^
	ADA	0.716	0.654	0.747	0.728
	GBM	0.747^a^	0.676	0.751^a^	0.727
Diseases of the musculoskeletal system and connective tissue	DT	0.68	0.690^b^	0.856	0.889^b^
	RF	0.719	0.734	0.909	0.882
	ADA	0.726^b^	0.739^a^	0.917^b^	0.915^a^
	GBM	0.751^a^	0.725	0.883^a^	0.9
Diseases of the respiratory system	DT	0.634	0.607	0.651	0.622
	RF	0.653	0.643	0.658	0.638
	ADA	0.663	0.667	0.639	0.655
	GBM	0.672^a^	0.675^a^	0.669^a^	0.667^a^

^a^Best performances for each disease.

^b^Major improvements in external validation.

**Table 4:** Weather and air quality predictors in W-score.

Disease groups	covariateName	covariateValue	CovariateMean	
			CovariateMean WithOutcome	CovariateMean WithNoOutcome
Diseases of the circulatory system	PM10	0.0016	12.59	13.13
	Rainfall	0.0011	2.22	2.32
	Humidity	0.0005	0.29	0.28
	Min Temperature	0.0006	0.65	0.59
	Max Temperature	0.0005	0.85	0.83
Mental and behavioral disorders	PM10	0.0016	12.36	13.24
	Rainfall	0.0012	1.95	2.30
	Humidity	0.0014	0.41	0.31
	Min Temperature	0.0008	0.67	0.58
	Max Temperature	0.0004	0.71	0.81
Diseases of the musculoskeletal system and connective tissue	PM10	0.0015	12.85	13.18
	Rainfall	0.0012	2.24	2.32
	Humidity	0.0008	0.31	0.30
	Min Temperature	0.0007	0.63	0.55
	Max Temperature	0.0007	0.86	0.84
Diseases of the respiratory system	PM10	0.0038	12.86	13.01
	Rainfall	0.0032	2.30	2.29
	Humidity	0.0005	0.31	0.29
	Min Temperature	0.0012	0.60	0.54
	Max Temperature	0.0036	1.01	0.97

Table 5 shows the details of the hyperparameter values used in this study.

**Table 5:** Summary of parameter values in each model.

Models	Parameters	Values	Parameter mean
DT	classWeight		“Balance” or “None”
	maxDepth	10	The maximum depth of the tree
	minImpuritySplit	10^−7^	Threshold for early stopping in tree growth. A node will split if its impurity is above the threshold, otherwise it is a leaf
	minSamplesLeaf	10	The minimum number of samples per leaf
	minSamplesSplit	2	The minimum samples per split
RF	Max depth	4, 10, 17	Max levels in a tree
	mtries	−1 = square root of total features, 5, 20	Number of features in each tree
	ntrees	500	Number of trees
ADA	Learning rate	1	Learning rate shrinks the contribution of each classifier by learning_rate. There is a trade-off between learningRate and nEstimators
	n estimators	4	The maximum number of estimators at which boosting is terminated
GBM	Learning rate	0.005, 0.01, 0.1	The boosting learn rate
	earlyStopRound	25	Stopping after rounds without improvement
	Max depth	4, 6, 17	Max levels in a tree
	minRows	2	Min data points in a node
	ntrees	100, 1000	Number of trees

**Figure 1:**
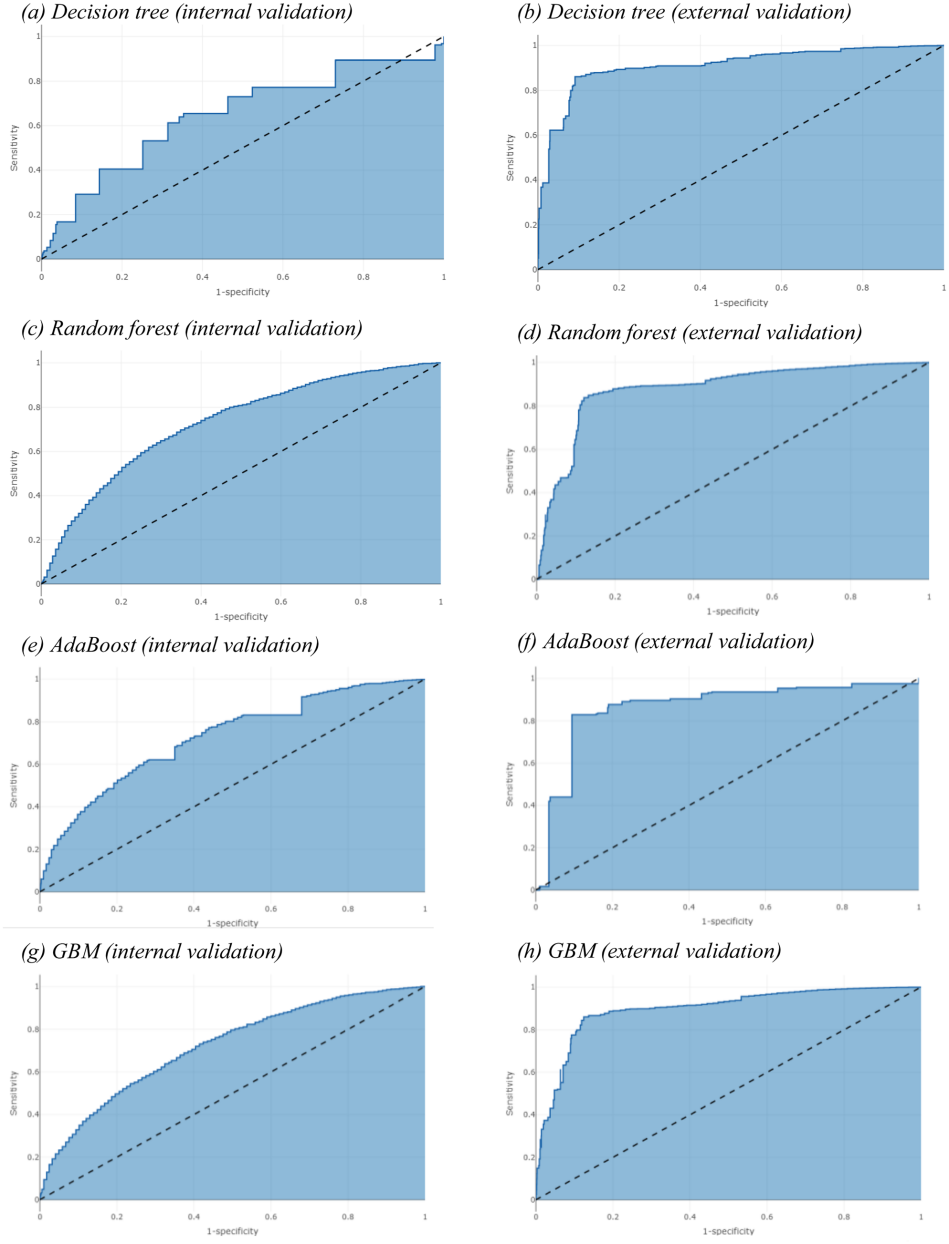
ROC curves for the validation of the Adaboost and decision tree models.

The receiver operating characteristic curves in Fig. 1 reflect the predictive model performances for the internal and external validation of the models based on clinical covariates and W-score in patients with diseases of the musculoskeletal system and connective tissue, respectively. The clinical covariate and W-score model exhibited the greatest AUC for both the internal and external validations in the musculoskeletal disease group.

## Discussion

We developed a 30-day unplanned hospital readmission prediction model based on OMOP-CDM transformed patient medical records and meteorological public data. We also obtained the weather and air quality records for the patients’ residence localities. Furthermore, we established a W-score for individual visits based on the Korean weather warning issuance criteria. In addition, we developed a model that can predict patient readmission when discharged by using weather forecast data directly from the clinical setting.

Many epidemiological studies have established an association between environmental factors and hospital readmissions^11,12,20,21^. However, few studies have examined the impact of environmental factors, such as ambient air pollution or climate, on hospital readmissions and the result of the health outcome using predictive analysis.

We developed a model to predict hospital readmission at the time of discharge based on patient-level clinical diagnosis and drug prescription data before discharge as well as the weather and air quality records for the patient’s residence locality. The variables used in the proposed model were designed based on diagnosis and drug information to make the model extensible, considering the standard term mapping issues that may arise in the process of converting electronic health record (EHR) data to OMOP CDM. This is because diagnostic and drug terminology do not differ significantly from the terminology used by most many hospitals.

The Korea Meteorological Administration (KMA) provides weather forecast information for a period from 3 to 10 days from the forecast date. If the KMA weather forecast and the hospital system are linked in the future, so if short-term weather forecast data for 7 days from the patient's discharge date are input to the developed readmission prediction model, the actual patient's readmission forecast information will be used for clinical decision making.

The performance of the proposed model for the respiratory disease cohort was lower than expected. Moreover, the performance of the proposed model for the musculoskeletal disease cohort demonstrated good scalability. These results are presumed to be due to the occurrence of readmission for acute events that require post-operative management, rather than hospitalization due to the occurrence of chronic diseases in tertiary hospitals. Many patients who needed trauma management after surgery were not hospitalized for a sufficient period. The results of a disease-specific predictive model can be observed in further studies based on our research.

We could not externally validate the proposed model across multiple organizations. However, the proposed model can be easily reintegrated when migrating to a different EHR, either as an embedded frame in the EHR or as a standalone CDM application. Furthermore, the proposed model can perform better using a sophisticated weather data function approach. Our research provides a basis for future applications of the proposed model to clinical settings, to manage visiting patients based on clinical and weather data.

In summary, providing a clinical basis for a patient’s future risk of readmission at the time of discharge will assist hospitals in developing a patient care plan in advance. We developed a model for predicting hospital readmission based on environmental factors. External verification of the model demonstrated that a high-accuracy model can be developed based on weather and air quality factors. Improving the accuracy of the readmission prediction model will help in establishing patient care plans and making clinical decisions at the time of discharge.

## Methods

### Study population and clinical data description

Our retrospective cohort study was conducted using OMOP-CDM-converted EHR data between January 1, 2017 and December 31, 2018 from SNUH and the Seoul National University Bundang Hospital (SNUBH) in the Seoul metropolitan area, South Korea. These hospitals have converted the EHR data over a 15-year period into the OMOP CDM.

We considered consecutive hospitalizations among adults over 65 years who were discharged alive and underwent at least one hospitalization or emergency-room visit during our study period. We focused on patients living in the Seoul metropolitan area, including the Gyeonggi Province in South Korea, to create prediction models that consider weather and environmental variables during the study period.

In addition, we categorized patients into subgroups based on weather- and environment-related diseases studied previously^20,22–27^. Patients diagnosed with mental and behavioral disorders (F00–F99), circulatory system diseases (I00–I99), respiratory diseases (J00–J99), and musculoskeletal system and connective tissue–related diseases (M00–M99) at discharge were included together in subgroups based on the International Classification of Diseases, 10th Revision.

The primary outcome of this study was 30-day unplanned hospital readmission. We referred to the Hospital-Wide All-Cause Unplanned Readmission (HWR) measure from Centers for Medicare & Medicaid Services (CMS)^28^. According to the HWR measure, CMS classified the planned readmissions into planned disease or treatment groups, including chemotherapy, organ transplant, and rehabilitation. All admissions other than the scheduled admissions were considered to be unscheduled visits.

**Figure 2:**
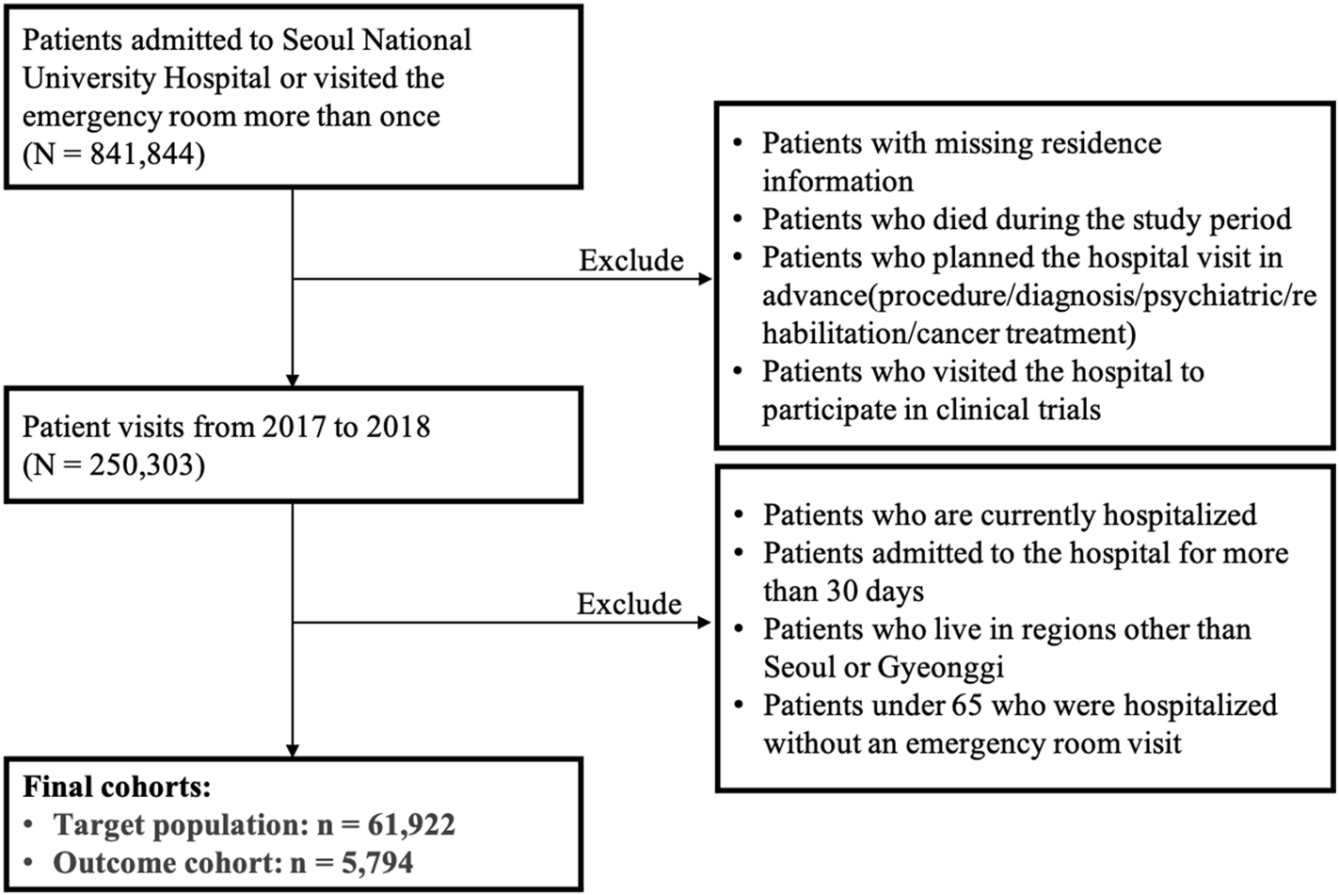
Study cohort design.

**Figure 3:**
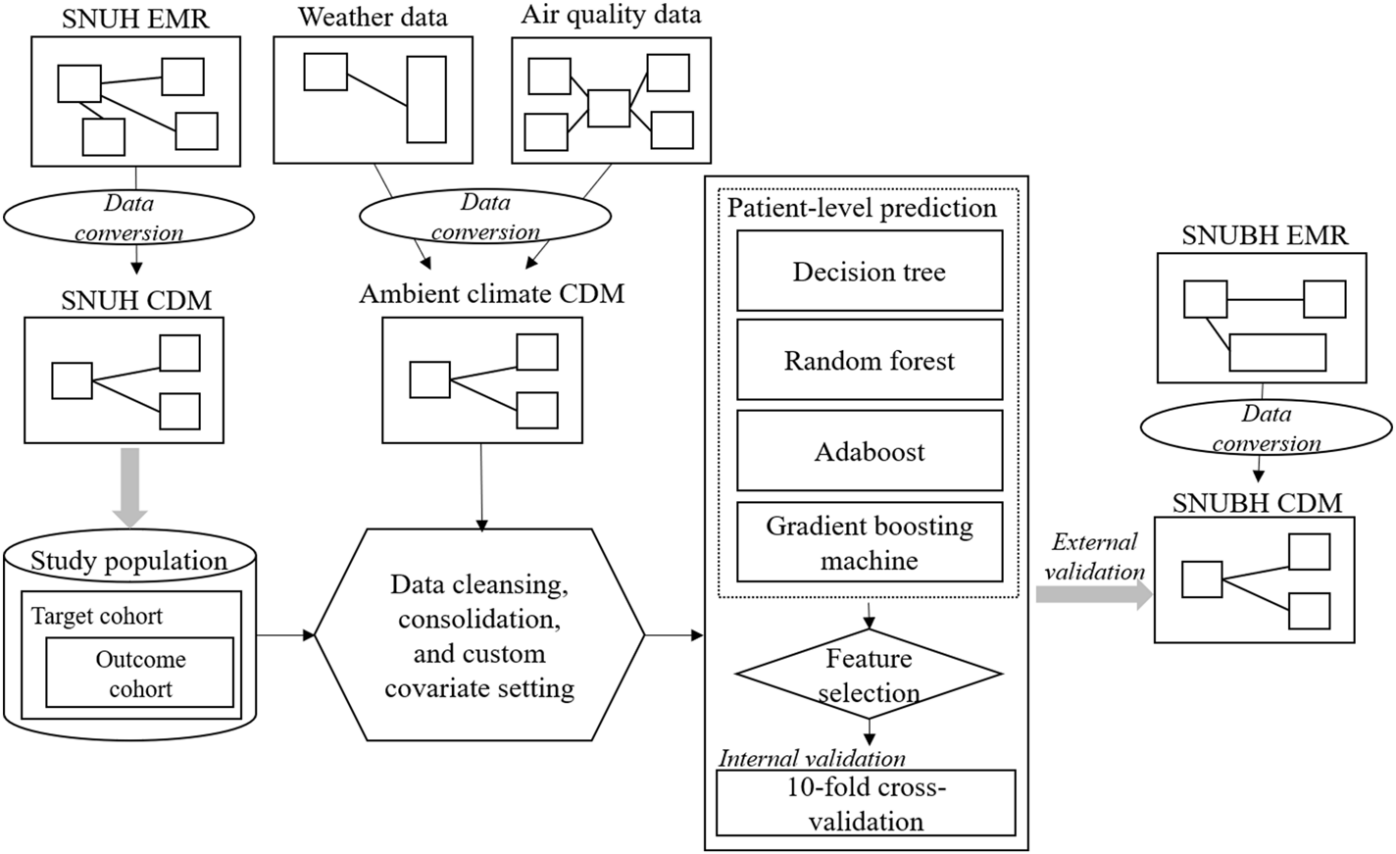
Overall methodology of the study.

Figure 2 illustrates the study cohort design derived using SNUH data, which are mainly used as the training dataset in our research. Figure 3 shows the overall study process in this research.

Clinical features, such as the gender of the patient, age of the subject on the index date, diagnosis conditions, drug exposures for patient medications, and the Charlson comorbidity index (Romano adaptation), were obtained using all conditions prior to the end of the readmission interval.

Diagnosis and drug prescription were used as clinical variables for individual patients. Moreover, each variable was extracted from the standardized CONDITION_ERA and DRUG_ERA in the CDM table as a higher concept of individual diagnosis and drugs. In OMOP CDM, a CONDITON_ERA data table is defined as the duration in which the patient is assumed to have a given condition^29^. The CONDITION_ERA table provided a chronological period of diagnosis. DRUG_ERA is defined as the duration in which the patient is assumed to be exposed to a particular active drug ingredient. The DRUG_ERA table provided successive periods of individual drug prescriptions combined following certain rules to produce continuous eras.

### Weather and air quality data

Weather and air quality data were derived from KMA’s weather data open portal (https://data.kma.go.kr) and the official website of the Korean Ministry Of Environment (MOE) (https://www.airkorea.or.kr/eng) ^30,31^.

Records of daily mean temperature (ºC), daily mean relative humidity (RH) percentage (%), and daily rainfall (mm) during the study period were obtained from the KMA website. The daily mean concentration of ambient particulate matter (PM in μg/m^3^), sulfur dioxide (SO_2_ in μg/m^3^), nitrogen dioxide (NO_2_ in μg/m^3^), and ozone (O_3_ in μg/m^3^) from all general monitoring stations were collected from the Air Korea website for the study period. The daily median was averaged across the data for any missing record on a particular day. KMA and Air Korea data needed to be preprocessed into postal zip codes owing to the varying levels of location information granularity. LOCATION_ ID in CDM DB has an address identifier based on the postal code address system. For example, LOCATION_ ID for Jongno-gu, Seoul does not match SNUH LOCATION_ ID and SNUBH LOCATION_ ID value. Therefore, it is necessary to first check the details of the LOCATION table of each institution CDM DB. Meteorological data of KMA and air environment data of Air Korea are recorded at each measuring station across the country. KMA data is divided into cities/metropolitan cities/provinces, and Air Korea data is based on a smaller unit, that is, the street address. Therefore, we first integrated KMA data and Air Korea data with the same granularity, and performed preprocessing by finding the postal code for the integrated address and matching it with the patient's residence address.

### W-score: weather and air quality scores for individual visits

We calculated a patient-level W-score based on weather and air quality data for each patient visit based on the patient’s residence locality. The score was derived using the KMA’s standards for special weather reports^32^. A special weather report refers to a forecast that calls attention to or warns against a serious disaster that is expected to occur because of a weather phenomenon. An “advisory” is issued if a disaster is expected because of a specific weather phenomenon, and a “warning” is issued if significant damage is expected. KMA issues weather reports on strong winds, wind waves, heavy rains, heavy snow, dry weather, storm tidal waves, earthquakes, cold waves, typhoons, yellow dust, and heat waves (Supplementary Table S1 and S2). Data such as the daily average particulate matter (PM10), maximum temperature, minimum temperature, relative humidity, and precipitation were used. Only PM10 was used among various atmospheric data, such as PM10, PM2.5, SO2, NO2, and O3, for calculating the W-score because there are many missing values of PM2.5 in the source data, and PM10 and PM2.5 have a multicollinear relationship.

W-scores of individual patient visits were calculated using weather conditions, such as fine dust warning, heat wave, cold wave, dryness, and heavy rain. The meteorological warning issuance criteria of the KMA were used for calculating W-scores for each element. We obtained the W-score by calculating the sum of the weather element–specific forecast values for 7 days from the discharge date so that the weather forecast data from KMA can be utilized at the time of patient discharge. Since its purpose is to predict readmission for this patient at the time of discharge, it is designed considering that weather forecast data for the next 7 days will be input and used for clinical decision-making.

### Model development

**Figure 4:**
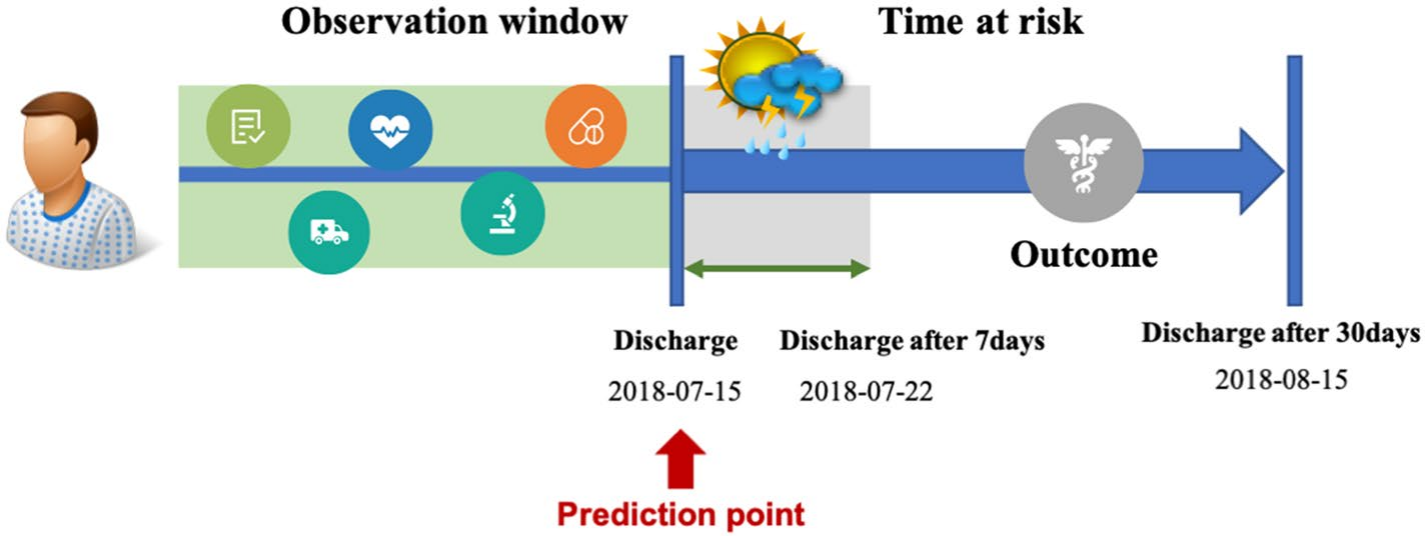
Prediction window.

The prediction model for re-admission within 30 days was developed to reflect variables such as clinical diagnosis and drug prescription prior to patient discharge date as well as to predict the occurrence of re-admission of the patient by considering the W-score for the weather forecast at the patient’s residence location after the discharge date (Fig. 4).

We developed tree-based machine learning models, namely, DT, random forest (RF), ADA, and gradient boosting machine (GBM)–based classifiers, based on the weather and air quality feature set using the patient-level prediction R package developed by OHDSI^19^. Models were trained and tested on SNUH data. All possible combinations of the hyper-parameters are included in a grid search using cross-validation on the training set. Ten-fold cross-validation is used to select the optimal hyper-parameter and internal validation. The hyper-parameters that lead to the best cross-validation performance will then be chosen for the final model. For our problem, we choose to build tree-based classifiers with several hyper-parameter values, as described in Table 5. Moreover, the models were externally validated using the SNUBH dataset. Each model performance was evaluated using the area under the receiver operating characteristic curve.

### Approval and consent waiver statement

This study was performed in accordance with the relevant guidelines and regulations of SNUH and SNUBH Institutional Review Board. As the data source was de-identified, this study was approved based on waivers of informed consent or exemptions by SNUH and SNUBH Institutional Review Board (SNUH IRB No: B-1504-296-302, SNUBH IRB No: X-1908-559-901).

## Supplementary Information

Supplementary Information 1.

Supplementary Information 2.
