# Supplementary material for: Thirty-day hospital readmission prediction model based on common data model with weather and air quality data
Source: Sci Rep. 2021 Dec 2;11:23313. doi: 10.1038/s41598-021-02395-9 (PMC8639801; doi:10.1038/s41598-021-02395-9)
Supplement: Supplementary file 1 — Supplementary Information 1. [file 41598_2021_2395_MOESM1_ESM.docx]

**Thirty-day hospital readmission prediction model based on common data model with weather and air quality data**

Borim Ryu^1,2^, Sooyoung Yoo^1*^, Seok Kim^1^, Jinwook Choi^2,3*^

^1^Office of eHealth Research and Business, Seoul National University Bundang Hospital, Seongnam, South Korea

^2^Department of Biomedical Engineering, College of Medicine, Seoul National University, Seoul, South Korea

^3^Institute of Medical & Biological Engineering, Medical Research Center, Seoul National University, Seoul, South Korea

**Corresponding authors:**

^*^Jinwook Choi, M.D.,Ph.D.

Department of Biomedical Engineering, College of Medicine, Seoul National University, 28 Yongon-dong Chongro-gu Seoul, Korea, 110-799

E-mail: jinchoi@snu.ac.kr; Tel: +82-2-2072-3421; Fax: +82-2-745-7870

^*^Sooyoung Yoo, Ph.D.

Office of eHealth Research and Businesses, Seoul National University Bundang Hosptital, 172, Dolma-ro, Bundang-gu, Seongnam-si, Gyeonggi-do 13605, Republic of Korea

E-mail: yoosoo0@snubh.org; Tel: +82-31-787- 8980

**Supplementary Table S1.** Weather advisory and warning issuance standards in Korea

| **Category** | **Criteria for issuing an weather advisory** | **Criteria for issuing an weather warning** | **W-score processing rule in this study** |
| --- | --- | --- | --- |
| Drying | When the effective humidity of 35% or less is expected to last for more than 2 days | When the effective humidity of 25% or less is expected to continue for more than 2 days | Days with humidity below 35% |
| Cold wave | In October-April, when one of the following is true:  1) When the morning minimum temperature falls by more than 10℃ than the previous day and is 3℃ or less and is expected to be 3℃ lower than the normal value  2) The morning minimum temperature is -12℃ or less for 2 days When it is expected to continue abnormally  3) When significant damage is expected due to a sudden low temperature phenomenon | In October-April, when one of the following is true:  1) When the morning minimum temperature falls by more than 15℃ than the previous day and is 3℃ or less and is expected to be 3℃ lower than the average value  2) The morning minimum temperature is -15℃ or less. When it is expected to last longer than a day  3) When significant damage is expected in a wide area due to a sudden low temperature phenomenon | 1) The day when the temperature difference between the minimum temperature and the previous day fell by more than 9 ℃  2) The minimum daily temperature is less than -10 ℃ |
| Heat wave | When the maximum daily temperature is 33℃ or higher is expected to last for 2 days or longer | When the maximum daily temperature is expected to last for more than 2 days when the maximum temperature is 35℃ or higher | Days when the maximum daily temperature is 33 ℃ or higher |
| Downpour | When 3 hours of rainfall is expected to be more than 60mm or 12 hours of rainfall is expected to be more than 110mm | When 3 hours of rainfall is expected more than 90mm, or 12 hours of rainfall is expected more than 180mm | It rained, or it didn't rain |

**Supplementary Table S2.** Ambient air quality standards for PM10 and PM25 in Korea

| **Category** | **Environmental standards**  **(daily average)** | **Forecast grade (daily average)** | | | |
| --- | --- | --- | --- | --- | --- |
|  |  | **Good** | **Moderate** | **Bad** | **Very bad** |
| PM 2.5 | 35 | 0 ~ 15 | 16 ~ 35 | 36 ~ 75 | 76 ~ |
| PM 10 | 100 | 0 ~ 30 | 31 ~ 80 | 81 ~ 150 | 151 ~ |
